# Simple low-cost 3D metal printing via plastic skeleton burning

**DOI:** 10.1038/s41598-022-11430-2

**Published:** 2022-05-13

**Authors:** Vladimir D. Burtsev, Tatyana S. Vosheva, Anton A. Khudykin, Pavel Ginzburg, Dmitry S. Filonov

**Affiliations:** 1 Center for Photonics and 2D Materials, Moscow Institute of Physics and Technology, Dolgoprudny, Russia 141700; 2 Telecom R&D Center, Moscow Institute of Physics and Technology, Dolgoprudny, Russia 141700; 3 School of Electrical Engineering, Tel Aviv University, 69978 Tel Aviv, Israel

**Keywords:** Electrical and electronic engineering, Design, synthesis and processing

## Abstract

Additive manufacturing of complex volumetric structures opened new frontiers in many technological fields, turning previously inconceivable designs into a practical reality. Electromagnetic components, including antenna and waveguiding elements, can benefit from exploring the third dimension. While fused deposition modeling (FDM) polymer printers become widely accessible, they manufacture structures with moderately low electromagnetic permittivities, compared to metals. However, metal 3D printers, being capable of producing complex volumetric constructions, remain extremely expensive and hard to maintain apparatus, suitable for high-end market applications. Here we develop a new metal printing technique, based on a low-cost and simple FDM device and subsequent electrochemical deposition. For testing the new method, we fabricated several antenna devices and compared their performances to standard printed FeCl_3_ etched board-based counterparts, demonstrating clear advantages of the new technique. Our new metal printing can be applied to manufacture electromagnetic devices as well as metallic structures for other applications.

## Introduction

Additive manufacturing enables exploring complex volumetric structures across variety of fundamental and applied disciplines^1^. The range of new capabilities allows reconsidering conventional approaches in mechanics^2–4^, thermal management^5^, medicine^6^, robotics^7^, electronics^8,9^, and many others applied areas, e.g.^10,11^ where novel architectures and material platforms can grant ever foreseen capabilities.

Hardware components, supporting wireless communication links, can also benefit from exploring volumetric geometries. Traditionally, planar architectures of radio frequency (RF) components, including waveguides and antennas, are integrated within printed electronic circuitry. This approach is favourable owing to well-established layer-by-layer lithographic fabrication. Functional 3D printing, however, allows exploring conceptually different designs with potentially better electromagnetic performances. While the surface equivalence principle suggests the ability to replace a volumetric realization with an impedance surface, enclosing the volume of the initial structure^12^, practical aspects play a role^13^, underlining real advantages of volumetric designs. Several additive manufacturing techniques were recently developed to create high-quality RF devices^14^. CNC milling^15,16^, laser direct structuring^17–19^, conformal printing of metallic inks^20,21^, ultrasonic wire mesh embedding^22^, and metal deposition through a mask on curved surfaces^23,24^ are among a series of the developed methods. Despite the proven performances of the beforehand mentioned techniques, those are designed per a specific task and yet can be considered as an ultimate solution in the field. On the other hand, fused deposition modeling (FDM) printers become available and are extremely low cost, making them the first choice in cases, when fast prototyping of volumetric structures is needed. FDM printers are compatible with a variety of polymer materials, including polylactic acid (PLA), acrylonitrile butadiene styrene (ABS), polyethylene terephthalate glycol (PETG), different alloys, polymer-nanostructures mixtures, and many others. Those plastics were already integrated within antenna devices (e.g.^25,26^). Furthermore, several polymer materials can be printed in parallel during manufacturing within a single session^27,28^. However, plastics are dielectrics with a relatively low electromagnetic contrast. Typically, the permittivity ranges between 2.5 and 3.5 at the 1–10 GHz band with the loss tangent of 10^−3^–10^−1^ for PLA^28^. Those numbers, though, depend on fabrication parameters, mainly on the polymer fill factor in a unit volume. Electromagnetic losses become dramatically high if conductive materials, e.g., graphene flakes, are mixed within polymer filaments. Here, loss tangent can approach unity, making those materials almost irrelevant for use in wireless communication devices. An ultimate solution for manufacturing volumetric RF devices is metal printing, e.g., performed with a direct metal laser sintering^29^. However, metal printers, nevertheless they provide high-quality standalone RF-contrast metal structures, remain extremely expensive, motivating the development of other approaches.

Here we demonstrate a simple and low-cost metal printing, based on a low-grade FDM technique. The new method is described first and then followed by a demonstration of several efficient electromagnetic devices, which are shown to outperform their conventional printed circuit board (PCB) counterparts.

## Results

### FDM metal printing

Metals with high RF conductivity allow obtaining superiorelectromagnetic performances. However, only a thin metal layer, having a thickness of several skin depths, governs the interaction^30^. Typically, several microns of copper is sufficient for the 1–10 GHz frequency range. It is worth noting that electroless plating can be used to cover non-conductive polymers, though this approach requires quite extensive chemical processing steps^31,32^. Another technique is electroplating, where electrochemical deposition is made on materials with a sufficient low-frequency conductivity. In our case, a structure’s 3D-printed skeleton serves as a cathode. For this purpose, initially isolating polymers should become conductive, which is done by introducing small particles. Filaments, made of PLA, mixed with graphene flakes (GPLA), will be used here. DC resistivity of this commercially available material (conductive PLA, 2.85 mm diameter, ‘Proto-pasta’) is about 0.1275 Ω m. It is worth noting that RF conductivity of this material is insufficient for practical applications. The permittivity of GPLA varieties between 52 and 15 at the 1–10 GHz frequency range, while the loss tangent is 0.75–0.87^33^. However, GPLA skeletons can serve as cathodes in electroplating. Micron to millimetre thickness layers of metals can be deposited on FDM-printed structures and serve GHz electromagnetic applications. However, electrochemically metalized GPLA remains as a substrate and, due to its relatively high permittivity, causes a high field concentration inside its volume, leading to moderately high losses. Those losses severely degrade antenna and waveguide performances, making the profitability of this approach questionable. Consequently, the removal of GPLA skeletons after their electroplating can drastically increase performances of 3D-printed electromagnetic devices. After performing this last skeleton removal step, freestanding metal structures are obtained. This new method will be described next.

**Figure 1:**
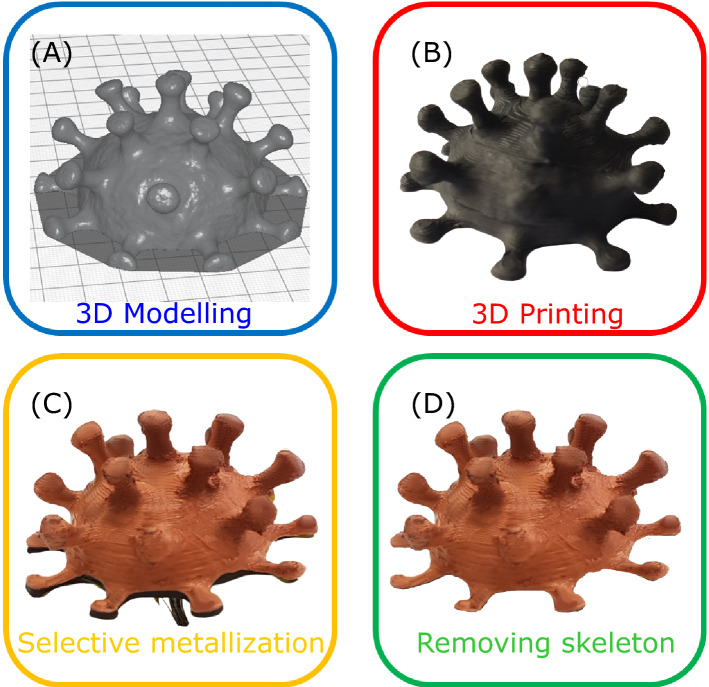
The proposed metal printing—the sequence of a free standing metal coronavirus prototyping. (**A**) 3D-modelling and slicing. (**B**) FDM 3D-printing of a conductive PLA (GPLA) skeleton. (**C**) Electroplating of the prototype’s surface. (**D**) Removal of the plastic skeleton.

The fabrication process is divided into several main steps. The first one is the printing a skeleton. BCN3D Sigmax printer was employed. BCN3D Cura 3.4.0 for slicing the model was used for the prototyping (Fig. 1A). After manufacturing the model, the next step is its post-processing. The structure was treated with a rag, wet with acetone or potentially, with another solvent (e.g., 1,2-dichloroethane, dichloromethane and others). This step allows smoothing roughness, occurred during the printing process own to finite thickness of FDM nozzles, 0.4 mm was used here. However, only small imperfections, e.g., cracks and bubbles, with sizes smaller than 0.5 mm can be efficiently diminished (Fig. 1B). The next stage is the electroplating. After the solvent preparation, the electroconductive part of the model was activated with copper sulfate solution. This makes the model surface more sensitive for further galvanization process due to increased ions adhesion^34^. To achieve a uniform deposition of metal ions on a surface, the later should be held at nearly uniform electric potential. While typical cathodes with initially high DC conductivity do not possess extra-challenges, GPLA skeletons with moderately high resistivity exhibit a significant voltage drop between adjacent electrodes. To improve the uniformity of the electric potential in this case, we distributed several electrodes along the sample. Those electrodes (copper wires) were isolated from the solution, otherwise the electrochemical deposition will primarily occur on contacts, leaving GPLA skeleton uncovered. After performing those technically uncomplicated steps, auxiliary electrodes of the structure were connected to the negative terminal of a current source (MATRIX MPS-3003L-3). A copper plate, serving as an ion source, was connected to the positive terminal. The galvanic bath contained 70:10:1 of water: Cu_2_SO_4_: sulfuric acid. The current in the galvanic circuit was calculated using the empirical ratio of 100 mA for every dm^2^ of electroconductive model surface, which is a compromise between the deposition quality and its rate. The deposition time is defined by a required metal thickness. The sample was removed from the galvanic bath and washed under running cold water at the end of the process (Fig. 1C). After the GPLA skeleton is metalized, the last stage is to remove the substrate. The melting point of typical polymers, used in FDM printing, is around 180-230ºC. Electrochemically deposited metal, however, sustains those temperatures. Hence, the skeleton can be removed in an oven, though minor oxidation of copper surfaces can be observed if air environment is used. Another option is to apply chemical removal or just burn the plastic with a gas jet, which we used here as the easiest option (Fig. 1D). As the result, the polymer skeleton is melted, whereas metal construction remains standalone—this is our proposed metal printing.

The characteristics of the resulting model are as follows: thickness of the metal layer is at least 0.5 mm, which permits the model to remain free standing after the skeleton removal, the overall size of the structure can reach dozens of centimeters along any direction (for our model the maximum is 11.8 cm), surface roughness is smaller than 0.1 mm and the surface coverage is quite uniform in all areas. The fabrication cycle is printing (3–8 h usually), post-processing (0.5–1 h), and electroplating (24–48 h).

### Electromagnetic performances of 3D-printed structures

To test the performances of the new fabrication technique, radio frequency identification (RFID) was chosen as an application. Rapid development of internet of things (IoT)^35^ and an emerging concept of internet of small things—IoST (e.g.^36^) motivates developing new miniature long-range tags with omni-directional responses (e.g.^37–39^). Here, efficient low-cost designs are essential for powering this application. RFID tags consist of an integrated circuit and an antenna, which, in many cases, governs the performances. Exploration of volumetric geometries can provide and advantage over conventional 2D designs.

**Figure 2:**
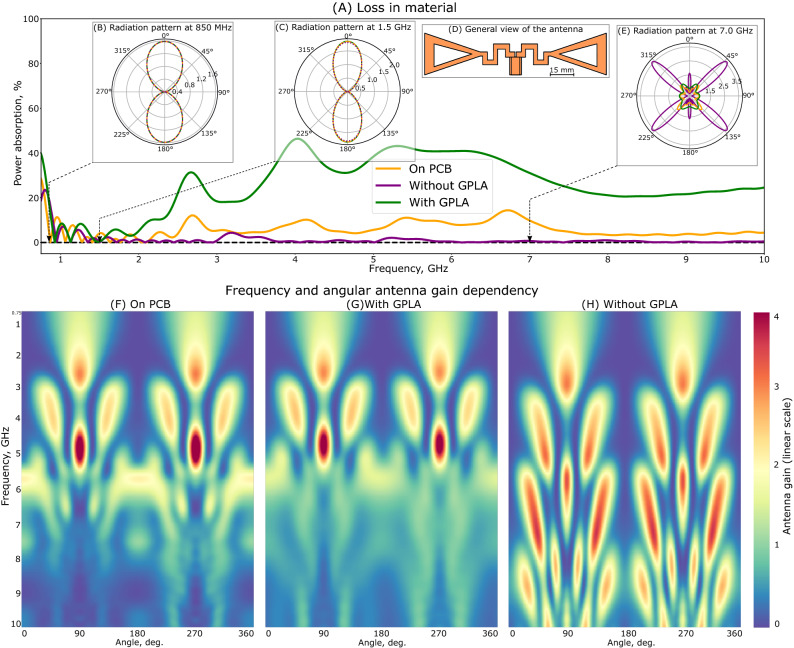
Numerical comparison between 2 antennas—with and without GPLA substrate. (**A**) Normalized absorbed power within the device (in %)—green line—with GPLA, purple—without GPLA. (**B**), (**C**) and (**E**) Radiation patterns at 850 MHz, 1.5 GHz and 7 GHz, respectively. (**D**) Antenna layout. (**F**), (**G**) and (**H**) Color maps of unwrapped radiation patterns (linear scale). Horizontal and vertical axes—angular and frequency dependences, respectively. Antenna gain is represented by color in linear scale.

Hereinafter, we will assess the impact of GPLA removal on antenna performances. A rather generic geometry, reported in^40^ (Fig. 2A, inset, panel D), will be used for the investigations. The structure is a dipole, considered in the frequency range from 750 MHz to 10 GHz, which captures both: the main resonant area (around 850 MHz, for which the structure was initially designed), and a non-resonant zone (1 to 10 GHz) where the antenna has a more complex radiation pattern and not necessarily impedance-matched. The rather known design was chosen for assessing performances of the new fabrication methodology versus existent standards. CST Microwave Studio was used for the analysis. Thickness of the GPLA substrate was taken as 2 mm uniform and conformal with the antenna. Figure 2A shows the absorbed power within the structure with and without GPLA skeleton. This parameter was calculated as a balance between four channels—power (i) entering launched into antenna port, (ii) total radiated (iii) back reflected to the port owing to the impedance mismatch, and (iv) absorbed. It is evident that GPLA removal significantly reduces the absorption along the entire frequency range. The difference is more pronounced at higher frequencies. In terms of radiation patterns, both configurations show a well-defined dipolar emission at lower frequencies (e.g., 850 MHz, Fig. 2B, and 1.5 GHz, Fig. 2C). However, at higher frequencies (7 GHz, Fig. 2E) low-loss freestanding metal antenna has quadrupole-like radiation pattern, while GPLA skeleton quenches the radiation quite significantly. Since the near-field localization grows with increasing the multipole number (e.g.^41,42^), the very pronounced difference between the radiation patterns is observed at high frequencies, where GPLA substrate demonstrates a severe absorption. While the structure is not resonant, it still has significant internal losses and cannot be used as a radiation element, underlining the clear advantage of the GPLA substrate removal. This general trend can be further seen while comparing panels (F), (G) and (H), where each horizontal cut of the colour map corresponds to the unwrapped radiation pattern. The GPLA skeleton lead to a significant blurring at higher frequencies. Maximal gain reaches 4 on the linear scale.

**Figure 3:**
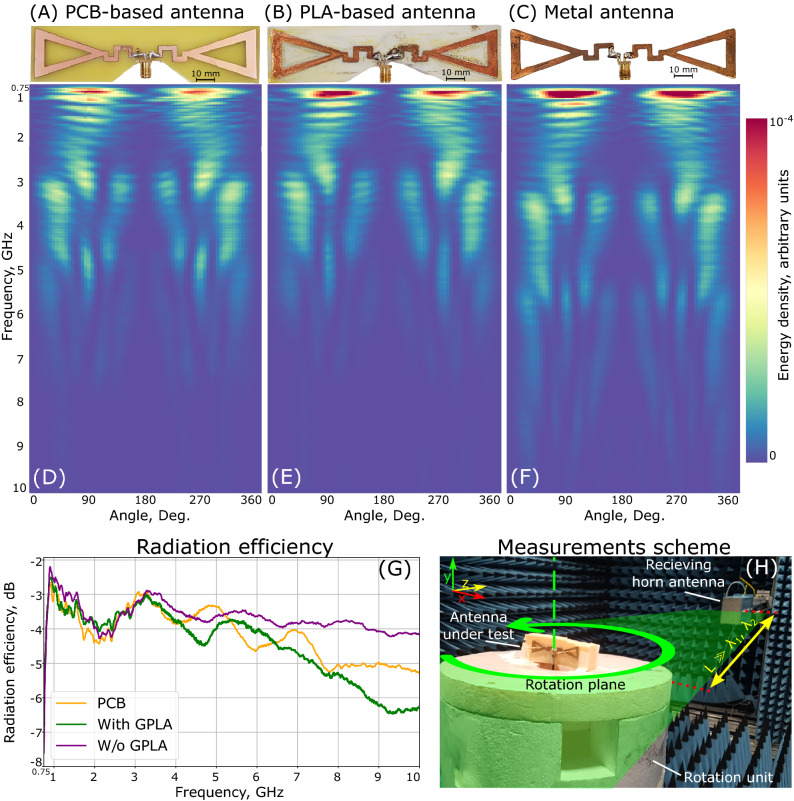
Experimental data representation. Comparison between (**A**) PCB-based, (**B**) GPLA substrate and (**C**) Free standing metal antennas. (**D**–**F**) Color maps of unwrapped radiation patterns (linear scale). Horizontal and vertical axes—angular and frequency dependences, respectively. Received energy is represented in linear scale, arbitrary units. (**G**) Radiation efficiency, as the function of frequency. PCB-based antenna—orange line, GPLA-based antenna—green, and metal antenna—purple. (**H**) Photograph of the experimental setup in an anechoic chamber.

To verify the beforehand made claims and assessments, experimental studies have been performed. Standard PCB design and fabrication were made for getting a reference sample. Figure 3 demonstrates the devices—PCB reference (panel A), 3D-printed antenna with GPLA skeleton (panel B), and standalone metal structure, which was obtained after GPLA removal (panel C). Antenna characteristics were acquired at an anechoic chamber (Fig. 3H). The antennas were connected to the Rohde & Schwarz RTO1024 vector network analyser (VNA) using a coaxial cable and mounted on an azimuth-rotary table opposite the measuring horn antenna (also connected to the same VNA). Polystyrene supports, being transparent to GHz waves, were used. The table was rotated between 0° and 360° with 1° steps. Complex transmission coefficients (S12) were obtained for the entire frequency range and for each angle. The colour maps (Fig. 3D–F) summarize the experimental results—horizontal lines are unwrapped angular radiation patterns at the sweep frequencies. Vertical lines represent the evolution of the radiation pattern with frequency. Metal freestanding antenna demonstrates the best performances, compared to both references. The advantage is more pronounced at higher frequencies, where both GPLA and FR4 (PCB material) have higher losses. Total radiation efficiencies of the samples were measured, and the results appear in Fig. 3G. Metal antenna outperforms the counterparts for all the frequencies within the band apart from several points, where the results fluctuated owing to parasitic reflections from the measurement apparatus.

## Discussion

A new simple and low-cost metal printing approach have been developed and its advantages in the field of additively manufactured electromagnetic devices were demonstrated. Our method is based on FDM printing of skeletons with the subsequent set of relatively straightforward post-processing operations. The process is summarized in the following five steps: (i) 3d printing of a skeleton with a conductive polymer, (ii) surface treatment for improving smoothness, (iii) auxiliary electrodes distribution on the skeleton, (iv) electroplating, and (v) skeleton removal. As the result of the process, freestanding metal structures can be obtained. The advantage of the substrate removal was analysed numerically and experientially, demonstrating significant improvement of antenna characteristics. In particular, high frequency loses were reduced by orders of magnitude compared to samples, where conductive polymer skeleton was present. The differences are much more pronounced at higher frequencies, where conductive polymers have looser performances. Furthermore, free-standing metal antennas, made by our new printing process, were shown to outperform standard PCB-based realizations, which also suffer from losses at frequencies above 5 GHz, if FR4 substrates are in use.

Though it is evident that our new method cannot compete with direct metal printing, based e.g., on laser sintering, on performances, it can provide sufficient solutions at extremely low cost. It is worth noting that fabricated structures had to be of an opened geometry for allowing the melted polymer flow out. The further advancement of this technology can allow creating more complex shapes and reveal their advantages in electromagnetic applications. Furthermore, there are quite a few efforts to 3D-print electronic circuitry—both passive and active elements. Given this capability developed, additive manufacturing of an antenna together with tunable electronics will become possible. The overall production cost in this case can drop significantly, making additive manufacturing of RF devices to be the referable first choice.
